# Diet and physical activity intervention preferences for young adult cancer survivors

**DOI:** 10.1007/s11764-025-01832-2

**Published:** 2025-05-30

**Authors:** Sylvia L. Crowder, Bihe Hu, Aasha I. Hoogland, Lisa M. Gudenkauf, Xiaoyin Li, Yvelise Rodriguez, Nathaly E. Irizarry-Arroyo, Laura B. Oswald, Brian D. Gonzalez, Brent J. Small, Andrew Galligan, Heather S. L. Jim, Marilyn Stern

**Affiliations:** 1 H. Lee Moffitt Cancer Center, Department of Health Outcomes and Behavior, 12902 Magnolia Drive, Tampa, FL 33612 USA; 2 School of Nursing, University of North Carolina at Chapel Hill, Chapel Hill, NC 33612 USA; 3 Department of Pediatrics, University of South Florida College of Medicine, Tampa, FL 33612 USA; 4 College of Behavioral and Community Sciences, University of South Florida, Tampa, FL 33612 USA

**Keywords:** Cancer, Cancer survivor, Diet, Empirical research, Nutrition, Patient preferences, Physical activity, Qualitative research, Young adult

## Abstract

**Objective:**

Patient preferences for diet and physical activity interventions likely vary by patient age at cancer diagnosis, gender, and ethnicity. Efficacious multiple health behavior interventions to improve diet and physical activity in young adult cancer survivors are critically needed. This study aimed to collect data to inform preferences for diet and physical activity interventions for young adult cancer survivors that could be modified, if needed, based on gender and ethnicity.

**Methods:**

Between May 2022 and April 2024, young adult cancer survivors between 20 and 30 years of age participated in an observational study to assess diet and physical activity habits and preferences. Participants completed a survey of diet and physical activity intervention preferences to be used for planning future intervention development. Descriptive statistics were calculated for multiple choice question responses. Intervention preferences for diet and physical activity were compared across self-reported gender (i.e., male vs. female) and race/ethnicity (i.e., Hispanic vs. other) using chi-square tests or Fisher’s exact tests (*α* = 0.05). Responses to open-ended questions were recorded and verified via quality assurance checks by a coauthor.

**Results:**

Study participants (*N* = 88) completed the preferences survey. Participants’ mean age at diagnosis was 23 years, 67% were female, 23% were from a racial/ethnic minority background, and common cancer types included thyroid (24%), lymphoid (19%), and sarcoma (15%). All participants had completed treatment at least 1 year prior to study enrollment (range 1–8 years). Overall, for a dietary intervention, most participants preferred remote-based (e.g., Zoom), one-on-one counseling with a registered dietitian beginning before treatment, and respondents expressed enthusiasm for free dietary coaching and virtual cooking classes. For a physical activity intervention, most participants preferred one-on-one, in-person sessions beginning before treatment, ideally within 1–15 miles from their home that would be free of charge. Participants reported interest in a variety of physical activities, including yoga, strength training, and cardiovascular-based activities (e.g., HIIT, interval training, swimming). There were few differences among gender and ethnicity.

**Conclusions:**

Results indicate that future diet and physical activity intervention development for young adult cancer survivors should focus on strategies to promote one-on-one telehealth dietary counseling and in-person physical activity training. Virtual cooking classes and cookbooks coupled with in-person fitness training were preferred methodologies for young adult cancer survivors in this study.

**Implications for Cancer Survivors:**

Study results highlight an opportunity to design a combined diet and physical activity intervention healthy lifestyle program for young adult cancer survivors that blend technology and personal contact.

## Introduction

The transition to young adulthood (i.e., age 20–30) is considered a significant psychosocial developmental stage for creating life-long behavioral habits [1, 2]. Lifestyle habits formed during this period tend to persist throughout the lifespan [3], and long-term health behaviors can affect health outcomes in older adulthood [4]. Compared to non-cancer peers, young adult cancer survivors are at increased risk for weight gain resulting from poor dietary intake and declines in physical activity [5]. Compared to peers without obesity, young adult cancer survivors with obesity experience earlier onset of cardiovascular disease [6] and are six times more likely to die from cardiovascular events, pulmonary diseases, or a subsequent cancer [7]. As a result of obesity-related adverse effects, young adult cancer survivors experience higher rates of late-term metabolic treatment effects [8], diabetes [9], and metabolic syndrome onset [8], with little improvement in trends in recent years [10–12].

Healthy lifestyle interventions combining diet and physical activity for young adult cancer survivors to prevent obesity-related late effects are clearly warranted. Yet, efficacious multiple health behavior studies to improve diet and physical activity in young adult survivors are lacking. The majority of studies incorporating diet and physical activity interventions include adolescents (i.e., 15–19 years of age) [13], recruit beyond the young adult age range (i.e., 39 years or older) [14–16], or focused on individual dietary components (e.g., fruit and vegetable intake) [17, 18] rather than enhancing overall diet quality. Further, the potential impacts of gender and ethnicity on diet and physical activity preferences are poorly understood, highlighting the need for more research to elucidate these influences [19, 20]. For instance, stereotypical gender roles could influence willingness to participate in physical activity interventions, as some exercises may be considered more “masculine” (e.g., weightlifting) or “feminine” (e.g., yoga). Additionally, young adult cancer survivors from racial and ethnic minority backgrounds likely have varying needs related to cultural background (e.g., food preferences, views on exercise). Understanding the ways in which gender and cultural characteristics may impact diet and physical activity preferences is important for enhancing the acceptability of diet and physical activity interventions.

The study aimed to assess diet and physical activity intervention preferences as reported by a diverse group of post-adjuvant treatment young adult cancer survivors. Our long-term goal is to inform a future framework for the development of a healthy lifestyle intervention for young adult cancer survivors from diverse ethnic and gender backgrounds with various cancer types, time since diagnosis, and treatments. By incorporating the patient voice, future diet and physical activity interventions hold vast potential for broader acceptability, while also allowing for personalization to best address individual preferences and needs.

## Methods

### Ethical approval

This study was performed in line with the principles of the Declaration of Helsinki and was approved by Advarra Institutional Review Board (IRB), the IRB of record for Moffitt Cancer Center (protocol #00000971). All participants were informed of the study purpose and procedures and were informed that study participation was voluntary, with participants retaining the right to withdraw at any time without explanation. All participants provided verbal informed consent prior to data collection. Patient health information was anonymized and assigned a number code to ensure confidentiality. No individuals are identifiable in any presentation of results.

#### Participant recruitment

Research coordinators screened the electronic medical records of potentially eligible young adult cancer survivors to determine eligibility and then contacted patients via phone, text message, e-mail, or in-person at a clinic visit to introduce patients to the study, allow time for questions, and solicit consent. Inclusion criteria included the following: (1) diagnosis of cancer between the ages of 19 and 29 years old; (2) currently between the ages of 20 and 30 years old; (3) at least 1 year post-adjuvant treatment; (4) ability to read/speak English; (5) ability to complete an online survey; and (6) ability to consume food orally. Exclusion criteria included women who were pregnant, as altered eating patterns may not represent their typical (pre-pregnancy) diet.

#### Measures

Participants completed a battery of survey measures assessing health history that included questions on age, gender, ethnicity, race, education, employment, income, and marital status. Clinical data were abstracted through medical charts and included treatment modality, cancer type, date of cancer diagnosis, and most recent body mass index.

#### Preferences

Participants completed a modified 18-item survey online, developed in collaboration with experts in diet and exercise oncology, to assess preferences for diet and physical activity interventions [21, 22]. Survey questions asked for dietary preferences regarding: (1) from whom they would like to receive dietary counseling (multiple choice; options were physician, nurse, registered dietitian affiliated at a cancer center, registered dietitian affiliated at a community center or health club, no preference), (2) preferred method for receiving dietary counseling (multiple choice; options for face-to-face, telephone, Zoom or another online visual communication tool, written material, DVD, interactive workbook, no preference), (3) size of counseling group (multiple choice; options were one-on-one session or group session), (4) importance of gender in dietary counseling sessions (multiple choice; options were preference for everyone to be the same gender (ex. all female) or combination of genders (ex. male and female), (5) willingness to pay for dietary counseling (multiple choice; options were $0, $1–10, $11–20, $21–30, $31–40, or > $40), (6) willingness to travel for dietary counseling (multiple choice, options were 0 miles, 1–15 miles, 16–30 miles, 31–45 miles, 46–60 miles, or > 60 miles), (7) ideal timing to start a dietary intervention program (multiple choice; options were before treatment, during treatment, immediately following treatment, 6 months after treatment, 1 year after treatment, or other). Two open-ended questions were included: (1) justify and explain the reasoning behind question 7 (ideal timing to start a dietary intervention program), and (2) topics of interest that participants would like to see incorporated into a dietary intervention program. Seven similar multiple-choice and two open-ended questions were asked regarding physical activity preferences. A $25 e-gift card was offered to all participants who completed the online survey.

#### Data analysis

Participant characteristics and multiple-choice survey responses were examined using means, standard deviations, frequencies, and percentages. Preferences for diet and physical activity interventions were presented as frequencies (%) and compared between genders and ethnicities using chi-square tests or Fisher’s exact tests when expected cell counts were < 5 (*α* = 0.05). All statistical analyses were performed using SAS version 9.4 (Cary, NC, USA). Responses to open-ended questions were recorded and then grouped based on themes and verified via quality assurance checks by a coauthor.

## Results

### Participant characteristics

Of 567 young adult cancer survivors screened for the study, 350 were ineligible, 280 did not meet the inclusion criteria, 64 were unreachable, and 6 had passed away. Of the 217 eligible participants, 100 consented to participate and 88 completed the preferences survey. Demographic and clinical characteristics are presented in Table 1. The mean age at diagnosis was 23 years old. Most participants were White, non-Hispanic, female, and had graduated from college. The majority were diagnosed with thyroid, lymphoid, sarcoma, and testicular cancer. All participants were at least 1 year post-adjuvant treatment completion (range 1–8 years).

**Table 1:** Demographic and clinical characteristics of young adult cancer survivors *N* = 88

Characteristic	Total *N* (%)
Age at diagnosis: mean ± SD [range], years	23.2 ± 2.5 [19–29]
Age at survey: mean ± SD [range], years	26.6 ± 2.4 [20–30]
Treatment completion: mean ± SD [range], years	2.5 ± 1.7 [1–8]
Female	59 (67.0)
Ethnicity: non-Hispanic	68 (77.3)
White: non-Hispanic	54 (79.4)
Black or African American: non-Hispanic	5 (7.4)
Asian: non-Hispanic	4 (5.8)
Other, more than one race: non-Hispanic	5 (7.4)
Ethnicity: Hispanic	20 (22.7)
White: Hispanic	14 (70.0)
Black or African American: Hispanic	1 (5.0)
Other, more than one race: Hispanic	5 (25.0)
College graduate	48 (54.5)
Annual household income* > $40,000	57 (73.1)
Not married**	66 (75.9)
Employed	64 (72.7)
Body mass index	
Healthy weight (BMI 18.5–24.9)	35 (39.8)
Overweight (BMI 25.0–29.9)	22 (25.0)
Obese (BMI ≥ 30)	31 (35.2)
Cancer treatment***	
Chemotherapy	59 (67.0)
Radiation	32 (36.4)
Immunotherapy	4 (4.5)
Hormonal	7 (8.0)
Surgery	57 (64.8)
Transplant	9 (10.2)
Tumor site	
Thyroid	21 (23.9)
Lymphoid	17 (19.3)
Sarcoma	13 (14.8)
Testicular	8 (9.1)
Leukemia	7 (8.0)
Breast	6 (6.8)
Brain	5 (5.7)
Other	11 (12.5)

^*^N = 78,

**N = 87

***Possible to answer more than one item per question

### Preferences

Table 2 presents preference survey responses among all participants, categorized by gender (female vs male) and ethnicity (Hispanic vs non-Hispanic). For a dietary intervention, most participants reported a preference for remote-based (e.g., Zoom), one-on-one counseling from a registered dietitian affiliated with a cancer center beginning before treatment, and participants expressed a strong preference for free dietary coaching. While both males and females preferred mixed-gender sessions, females were more open to accepting same-gender sessions compared to males (37.9% vs 14.3%). While both Hispanic and non-Hispanic participants preferred free dietary counseling ($0), Hispanic participants demonstrated a greater willingness to pay, with 21.0% willing to pay $30 or more per month compared to 5.9% of non-Hispanic participants.

**Table 2:** Young adults’ cancer survivors’ preferences for diet and physical activity interventions

Question	*N* (%) total (*N* = 88)	*N* (%) female (*N* = 59)	*N* (%) Hispanic (*N* = 20)
Dietary intervention preferences			
From whom would you most like to receive dietary counseling?			
Physician	3 (3.4)	3 (5.1)	1 (5.0)
Nurse	1 (1.1)	1 (1.7)	0 (0.0)
Registered dietitian affiliated at a cancer center	43 (48.9)	31 (52.5)	10 (50.0)
Registered dietitian affiliated at a community center or health club	6 (6.8)	4 (6.8)	2 (10.0)
No preference	35 (39.8)	20 (33.9)	7 (35.0)
How would you like to receive diet counseling?			
Face-to-face	14 (15.9)	11 (18.6)	1 (5.0)
Telephone	8 (9.1)	2 (3.4)	2 (10.0)
Online visual communication tool (ex. ZOOM)	22 (25.0)	16 (27.1)	8 (40.0)
Written material	17 (19.3)	14 (23.7)	1 (5.0)
DVD	1 (1.1)	1 (1.7)	0 (0.0)
Interactive workbook	5 (5.7)	4 (6.8)	1 (5.0)
No preference	21 (23.9)	11 (18.5)	7 (35.0)
What size would you prefer the dietary counseling group?			
One-on-one	73 (83.0)	50 (84.8)	16 (80.0)
Group session	15 (17.0)	9 (15.2)	4 (20.0)
If we offered group-based dietary sessions, how important is gender?*			
I prefer everyone to be the same gender as me (ex. female)	26 (30.2)	22 (38.0)^+^	4 (21.0)
I prefer a combination of genders (ex. male and female)	60 (69.8)	36 (62.0)	15 (79.0)
How much are you willing to pay for dietary counseling per month?**			
$0	38 (43.7)	24 (41.4)	7 (36.8)
$1–10	13 (15.0)	10 (17.2)	5 (26.3)
$11–20	15 (17.2)	11 (19.0)	0 (0.0)
$21–30	13 (15.0)	9 (15.5)	3 (15.8)
$31–40	5 (5.7)	1 (1.7)	3 (15.8)
> $40	3 (3.4)	3 (5.2)	1 (5.3)
How far are you willing to travel for dietary counseling?			
0 miles	21 (23.9)	11 (18.6)	3 (15.0)
1–15 miles	41 (46.5)	23 (44.1)	10 (50.0)
16–30 miles	21 (23.9)	17 (28.8)	6 (30.0)
31–45 miles	2 (2.3)	2 (3.4)	0 (0.0)
46–60 miles	1 (1.1)	1 (1.7)	0 (0.0)
> 60 miles	2 (2.3)	2 (3.4)	1 (5.0)
What is the ideal timing to start a dietary intervention program?			
Before treatment	25 (28.4)	17 (28.8)	5 (25.0)
During treatment	14 (15.9)	12 (20.3)	1 (5.0)
Immediately following treatment	13 (14.8)	11 (18.7)	6 (30.0)
6 months after treatment	7 (8.0)	3 (5.1)	2 (10.0)
1-year after treatment	8 (9.1)	5 (8.5)	2 (10.0)
Other	21 (23.8)	11 (18.6)	4 (20.0)
Physical activity intervention preferences			
From whom would you like to most receive physical activity counseling?			
Physician	12 (13.6)	8 (13.6)	3 (15.0)
Nurse	1 (1.1)	1 (1.7)	1 (5.0)
Exercise specialist affiliated with a cancer center	18 (20.5)	14 (23.7)	5 (25.0)
Exercise specialist affiliated with a community center	13 (14.8)	8 (13.6)	2 (10.0)
Cancer patient or survivor	1 (1.1)	1 (1.7)	1 (5.0)
No preference	43 (48.9)	27 (45.7)	8 (40.0)
How would you like to receive physical activity counseling?			
Face-to-face	29 (33.0)	22 (37.3)	7 (35.0)
Telephone	8 (9.1)	2 (3.4)	1 (5.0)
Online visual communication tool (ex. ZOOM)	11 (12.5)	10 (17.0)	4 (20.0)
Written material	10 (11.4)	7 (11.9)	3 (15.0)
DVD	0 (0.0)	0 (0.0)	0 (0.0)
Interactive workbook	4 (4.5)	4 (6.7)	1 (5.0)
No preference	26 (29.5)	14 (23.7)	26 (20.0)
What size would you prefer the physical activity group?**			
One-on-one	55 (63.2)	35 (59.3)	13 (65.0)
Group session	32 (36.8)	24 (40.7)	7 (35.0)
If we offered group-based physical activity sessions, how important is gender?**			
I prefer everyone to the same gender as me (ex. female)	30 (34.5)	25 (42.4)	6 (30.0)
I prefer a combination of genders (ex. male and female)	57 (65.5)	34 (57.6)	14 (70.0)
How much are you willing to pay for physical activity per month?			
$0	38 (43.2)	22 (37.3)	8 (40.0)
$1–10	11 (12.5)	9 (15.3)	3 (15.0)
$11–20	17 (19.3)	11 (18.6)	1 (5.0)
$21–30	10 (11.4)	8 (13.6)	4 (20.0)
$31–40	7 (8.0)	4 (6.8)	3 (15.0)
> $40	5 (5.6)	5 (8.4)	1 (5.0)
How far are you willing to travel for physical activity counseling?			
0 miles	20 (22.8)	9 (15.2)	2 (10.0)
1–15 miles	42 (47.7)	27 (45.8)	12 (60.0)
16–30 miles	24 (27.3)	21 (35.6)	6 (30.0)
31–45 miles	1 (1.1)	1 (1.7)	0 (0.0)
46–60 miles	1 (1.1)	1 (1.7)	0 (0.0)
> 60 miles	0 (0.0)	0 (0.0)	0 (0.0)
What is the ideal timing to start a physical activity intervention program?			
Before treatment	23 (26.1)	16 (27.1)	4 (20.0)
During treatment	7 (8.0)	5 (8.5)	0 (0.0)
Immediately following treatment	15 (17.0)	11 (18.6)	2 (10.0)
6 months after treatment	11 (12.5)	7 (11.9)	6 (30.0)
1-year after treatment	11 (12.5)	8 (13.6)	4 (20.0)
Other	21 (23.9)	12 (20.3)	4 (20.0)

^*^N = 86

**N = 87

^+^A statistically significant difference (*p* < 0.05)

For a physical activity intervention, most participants reported a preference for free one-on-one, face-to-face sessions beginning before treatment, ideally within 1–15 miles from their home. Gender differences emerged across multiple dimensions, including gender importance, preferred counseling methods, and travel distance. As with dietary coaching, both males and females preferred mixed-gender sessions, but females showed 3.4 times a higher likelihood of accepting same-gender sessions than males (42.4% vs 17.9%). For counseling methods, most participants preferred either face-to-face counseling or had no preference; females more often chose face-to-face (37.3% vs 24.1%), online communication (17.0% vs 3.4%), and interactive workbook (6.8% vs 0), but males more frequently selected “no preference” (41.4% vs 23.7%) and telephone-based counseling (20.7% vs 3.4%). For travel distance, both males and females most commonly preferred 1–15 miles; however, males were much more likely to limit their travel to 15 miles or less (89.7% of males vs 61.0% of females), whereas females showed more willingness to travel longer distances.

### Open-ended responses

The responses to the open-ended questions asking for justification of participants’ choice of ideal timing and topics were grouped into categories. Most participants preferred a dietary intervention starting before treatment, citing the need for more dietary guidance prior to treatment and greater clarity on what to eat during treatment. For example, one participant noted that “it is important to start dietary interventions as soon as you can” (#0003, female, 21 at diagnosis), and another indicated that initiating a diet intervention before treatment “…could also reduce the stress of having to make so many changes at once (during treatment)” (#0014, female, 20 at diagnosis). Similarly, one participant stated:

There are a few tips and tricks that I learned along the way but I had to Google anything that I wanted to know; I didn’t feel that there was enough information provided on how much treatment would affect me. Had I adjusted my diet before treatment, I would have felt 10x better and my transition into radiation and chemotherapy wouldn’t have affected me as badly as it initially did. It’s also easier to make the diet adjustments before the pallet changes during chemo and it helps to build healthy habits throughout treatment. (#0062, Male, 23 at diagnosis)

When asked which topics to incorporate into a dietary intervention, main themes included cooking classes, cookbooks, recipes, and reading nutrition labels. One participant noted they “…would love some recommendations on cookbooks and ingredients to incorporate [into recipes] that have helped other cancer survivors (from easy meals to complicated ones)” (#0095, male, 22 at diagnosis) and another stated that “kitchen cooking classes would be great, thinking about cooking on a budget, also thinking about portion size and not making huge amounts of a meal, it seems that most things are targeted to a family when many young adults may have a small family or live alone, so considering how to scale recipes would be great” (#0110, female, 20 at diagnosis).

Consistent with preferences for the timing of diet interventions, participants reported a preference for starting physical activity interventions before treatment. One participant acknowledged that it could be “…hard to get motivated to do anything when feeling unwell,” and starting before treatment “…would allow the team to be better able to adjust it as needed without also overwhelming the patient in the middle of treatment” (#0082, female, 19 at diagnosis). Another participant, who was a former personal trainer, noted that “all healthy habits should be built before you start treatment,” and “it’s a huge accomplishment to be able to finish the day and say that I did a full workout whether that was just walking around the block or doing some bodyweight squats. The doctors also told me that my recovery time was much faster than most patients because of my athletic and extremely active background” (#0062, male, 23 at diagnosis). Other participants indicated that starting a physical activity regimen prior to treatment would have helped prevent substantial muscle loss and would have helped with symptom management.

When asked which topics to incorporate into a physical activity intervention, main themes included yoga, resistance training, and cardiovascular activities. One participant indicated that they would like “yoga and cardio most importantly, and a course on how to responsibly start lifting again that takes into account how weak cancer treatment leaves the body. It is very frustrating feeling weak after chemotherapy so I think that it would be nice to help people who want it to regain their strength” (#0117, male, 19 at diagnosis). A second participant stated “doable workouts for your type of body…some people may only be able to go for a 10-min walk while others may be able to break a sweat. Have variety and options to choose from” (#0053, female, 20 at diagnosis). A third participant discussed the importance of having one-on-one sessions that were personalized, describing the need for “tailored exercises for different limits (i.e., some people can’t walk during treatment so do exercises while sitting down, regaining muscle back, etc.)” (#0106, female, 21 at diagnosis).

## Discussion

Diet and physical activity are promising non-pharmacological, non-invasive, and cost-effective interventions associated with several health benefits [23–26]. This study reported young adult cancer survivors’ preferences for diet and physical activity interventions. Notable findings were that most survivors reported preferring Zoom-based dietary counseling and in-person physical activity sessions, both starting prior to treatment. Participants expressed particular interest in virtual cooking classes with recipes from cookbooks, as well as in-person yoga, resistance training, and cardio-based physical activity interventions.

Previous research has suggested that racial/ethnic minority young adult survivors, particularly Hispanic survivors, have varying intervention preferences. For instance, studies have shown that adults from African American and non-Hispanic White backgrounds report a preference for digital health interventions [27–30] while other studies indicate that adults from Hispanic backgrounds prefer in-person interventions [31, 32]. Our results, however, suggest that young adults from both non-Hispanic and Hispanic backgrounds prefer Zoom-based dietary and face-to-face physical activity interventions. Combining remote-based dietary counseling with an in-person physical activity intervention could help increase research accrual and retention and could ultimately improve intervention participation in clinical settings, as this combination caters to individual patient preferences, while providing continuous support to reinforce behavior change. Remote dietary counseling and cooking classes offer a sense of connectedness without the added logistical burden of travel, while in-person physical activity training allows for tailored feedback and immediate adjustments to physical exercises and performance goals.

While both males and females reported an overall preference for mixed-gender diet and physical activity intervention sessions, females were significantly more likely to prefer same-gender diet and physical activity sessions compared to males. The preference for mixed-gender interventions is similar to previous work exploring preferences for supportive care interventions, in which most young adult cancer survivors preferred groups with mixed genders (41%) or no preference (41%) [33]. Future intervention work should consider mixed-gender diet and physical activity interventions.

Most participants preferred no-cost diet and physical activity interventions. This is consistent with previous U.S. research showing that young adult cancer survivors bear disproportionate financial toxicity concerns compared to adult cancer survivors, including worse material hardship, coping resource depletion, and psychological burden [34]. Given that medical nutrition therapy is often not covered for oncology populations—despite their substantial nutritional needs—there is a compelling case for advocating changes in insurance benefit design to improve access to care. Changes in insurance plan benefit designs and insurance coverage policies to ensure early preventive and therapeutic access to care could help address cancer-related financial stress, meet young adult survivors’ needs for dietary and physical activity services, and improve cancer-related outcomes overall [21, 35]. Additionally, future work could explore tiered service delivery models, where basic components of the intervention (e.g., peer support, education, self-guided tools) are made freely accessible, while more individualized services are supported from sliding scale fees, research grants, or partnerships with community organizations.

This study offers an important contribution to the existing body of literature for dietary and physical activity interventions by amplifying the voice of young adult cancer survivors [13–18] and identifying specific, actionable preferences for future interventions. A strength of the study was our ability to consent 46% of eligible participants, in which 41% completed the diet and physical activity preferences survey. A recent literature review assessing strategies for recruitment and retention of cancer survivors between 15 and 39 years of age found that survivor recruitment rates for this population are generally lower than other age groups, with enrollment ranging around 17% [36]. Study limitations should also be noted. Our sample size is small but offers novel, critical information on dietary and physical activity intervention preferences in our patient population (e.g., young adult cancer survivors between 20 and 30 years old). Furthermore, study evaluation methods were descriptive, and future studies may benefit from additional qualitative probing to further increase the robustness of research findings. Finally, generalizability of results may be limited by the survey’s response rate, risk of selection bias, sample sociodemographic characteristics (e.g., mostly female, highly educated), and geographical location.

## Conclusions

Young adult cancer survivors reported preferences for no-cost diet and physical activity interventions beginning prior to treatment. Specific to diet interventions, young adults preferred remotely delivered dietary counseling from a registered dietitian affiliated with a cancer center, highlighting the importance of insurance reimbursement for oncology nutrition services. Registered dietitians are uniquely poised to lead the “food as medicine” initiative through virtual nutrition counseling and educational cooking classes, offering a promising means of managing and improving numerous cancer outcomes [37]. Specific to physical activity interventions, young adults reported a preference for one-on-one, face-to-face supervised sessions. Planning for future diet and physical activity interventions for young adult cancer survivors should focus on strategies to promote telehealth dietary counseling or virtual cooking classes, along with in-person fitness training.

## Data Availability

Data is available from the corresponding author upon reasonable request.
